# 10 years of EJNMMI Research: an overview

**DOI:** 10.1186/s13550-021-00781-y

**Published:** 2021-04-15

**Authors:** Angelika Bischof Delaloye

**Affiliations:** CHUV-FBM University of Lausanne, Lausanne, Switzerland

Ten years ago, in 2011, the first articles were published in EJNMMI Research. When Ignasi Carrió, at the time Editor-in-Chief of EJNMMI, together with Ute Heilmann, Editorial Director Clinical Medicine, Springer-Verlag Heidelberg, presented the project to launch a new European journal of nuclear medicine as a companion journal to the prestigious EJNMMI, the reaction of the EJNMMI Editorial Board was mixed. This even more that the new journal would be an open access journal. Many members of the Board feared that the need to pay article processing charges would prevent authors from submitting manuscripts; others were not sure about the respective roles of EJNMMI and EJNMMI Research and worried about potential overlaps. However, the response of the nuclear medicine scientific community to the new publication platform quickly overcame these concerns. Furthermore, the fact that most funding agencies currently ask scientific results to be made widely available and support open access contributed to the development of the journal.

After EJNMMI Research, other open access journals, EJNMMI Physics, EJNMMI Radiopharmacy and Chemistry and the European Journal of Hybrid Imaging, have been launched by Springer Nature as additional platforms of scientific exchange within the “EJNMMI Family”, in close connection and with the effective support of the EANM.

In 2011, EJNMMI Research published 37 manuscripts, in 2020 this number increased by a factor of four to 155. From the beginning, we decided to be selective, so the rejection rate was close to 40% already in the first years; due to the increased number of submissions, this percentage now represents the approximate acceptance rate. Submissions are received from all over the world, in 2020 mainly from Europe (162) and Asia (157). The Americas submitted 46, Africa 2 and Australia 13 manuscripts. The country with the largest number of submissions was China (78). All these manuscripts were read and evaluated by the editor first to find appropriate reviewers. This task is not always easy, especially when some very specific molecular targets are studied in preclinical settings. In 2020, 2045 reviewers from Europe (1235), the Americas (548), Asia (205), Australia (49) and Africa (8) gave their invaluable support to the journal by evaluating manuscripts and assisted me in selecting the most remarkable publications. Here, I want to express my gratitude for the excellent reviews produced, most often well within the short time limit of 14 days, and the constant availability for a re-review of revised manuscripts. The large majority of reviews was exhaustive, benevolent and reliable. Even when reviewers proposed to reject the manuscript, excellent comments have usually been provided to the authors to assist them in improving their work. Consequently, many authors expressed their gratitude and appreciation of the reviewers’ comments.

In these ten years, the aim was to favor novelty and quality of the scientific work to be published rather than hunting the impact factor. Some results still preliminary but encouraging were published to make them available to the scientific community, to incite further research and discussion. The submission of studies that did not provide the expected results was also encouraged. Negative results, if clearly recognized as such, are as important as positive ones; they should, therefore, be known by the scientific community and not disappear in a drawer or a forgotten file on a personal computer. Their publication helps others to avoid spending time, energy and money in studies that have already been proven unsuccessful. Knowing the lack of success of an experiment, new ways might be designed to circumvent or overcome potential drawbacks and consequently advance science.

Today EJNMMI Research has found its place among the international journals publishing manuscripts in radiology and nuclear medicine. It is largely known, accessed and quoted. For 2019, the last available complete data, the Scopus CiteScore ranked EJNMMI Research 65th of 284 journals in the field of radiology, nuclear medicine and imaging, and at the 77th percentile (https://www.scopus.com/sourceid/21100235610?origin=sbrowse).

Now, time has come to move the journal to the next level and to hand the responsibility of the journal over to a new Editor-in-Chief. I am happy that Wim Oyen accepted this task and hope that he will find as much satisfaction and pleasure as I did, while I have no doubt on his success in further developing the journal. I look forward to seeing the journal take a new momentum.

Finally, I wish to thank all those who have trusted and supported me in the task of Editor-in-Chief, first of all Ignasi Carrió and Ute Heilmann, who invited me to participate in founding and developing the first open access companion journal to the EJNMMI. My gratitude goes to all persons with whom I had the pleasure to work at Springer Nature and Biomed Central; in particular, I wish to thank Sabine Ben Ghechir for her continuous encouragement and support. I am also thankful to all those who were successively in charge of the journal at the Editorial Office, who assisted me in the daily editorial tasks for more than ten years. Special thanks go to the Editorial Board and Associate Editors for advice and help with complex decisions.

Above all I would like to express my thanks and appreciation to the authors who have supported the journal by submitting their manuscripts from the very beginning and also to the readers of EJNMMI Research. They are the ones who finally made and continue to make the success of the journal.

